# Effect of Microwave on Protein Conformations and Enzymatic Reactions

**DOI:** 10.3390/molecules31111843

**Published:** 2026-05-27

**Authors:** Fumihiro Kayamori, Kenji Usui

**Affiliations:** 1 Faculty of Frontiers of Innovative Research in Science and Technology (FIRST), Konan University, Chuo-ku, Kobe 650-0047, Hyogo, Japan; kusui@konan-u.ac.jp; 2 Research Institute for Nanobio-Environment and Non-Ionizing Radiation (RINNIR), Konan University, Chuo-ku, Kobe 650-0047, Hyogo, Japan; 3 Beyond5G Donated Lectures, Konan University, Higashinada-ku, Kobe 658-8501, Hyogo, Japan

**Keywords:** microwave, enzyme, protein, enzyme conformation, enzymatic reaction

## Abstract

Microwave (MW) technology has attracted considerable attention as a promising approach for environmentally benign chemical processes owing to its high energy efficiency and potential for process simplification. Similarly, enzymatic reactions, which are characterized by high substrate specificity and mild reaction conditions, are indispensable technologies in both academic research and industrial applications and are widely recognized as representative green chemistry processes owing to their feasibility in aqueous media and the suppression of byproduct formation. The integration of enzymatic reactions with MW technology, referred to as MW-assisted enzymatic reactions, is therefore regarded as a promising research area. Enzymes, which are predominantly proteins and are structurally more complex than peptides, function as key catalytic entities in diverse biochemical reactions. Investigating the interactions between MWs and proteins and understanding how these interactions induce structural changes in protein molecules areessential for elucidating the effects of MW irradiation on enzymatic reactions. This review focuses on the effects of MW irradiation on protein conformations and enzymatic reactions. The insights presented herein are expected to contribute to a deeper understanding of MW-assisted enzymatic processes and the rational design of future sustainable reaction systems.

## 1. Introduction

The development of novel reaction technologies based on the principles of green chemistry is in high demand for a sustainable society. In particular, microwave (MW) technology, which offers high energy efficiency and enables process simplification, has attracted considerable attention as a promising approach for environmentally benign chemical processes. MWs are electromagnetic waves with frequencies ranging from 300 MHz to 300 GHz. Since MW-assisted organic synthesis was first reported in 1986 [1,2], MWs have been increasingly applied in the synthesis of a wide variety of organic compounds [3,4,5,6,7,8] and inorganic materials [9,10,11], and their range of applications continues to expand.

Enzymatic reactions, characterized by high substrate specificity and mild reaction conditions, have become indispensable for academic research and industrial applications [12,13,14,15]. Owing to advantages such as the feasibility of reactions in aqueous media and the suppression of byproduct formation, enzymatic reactions are widely recognized as representative reaction systems that embody the principles of green chemistry. Therefore, the combination of enzymatic reactions with MW technology, namely MW-assisted enzymatic reactions, is regarded as a promising research area. Despite the increasing number of studies on MW-assisted enzymatic reactions in recent years [16,17], these findings remain fragmented across disciplines. Moreover, MW-specific effects on enzymatic reactions that cannot be explained solely by an increase in bulk temperature have been suggested [18]. Such phenomena have been discussed in terms of localized temperature heterogeneities at the protein–water interface, which may arise from the selective interaction of MW fields with polar water molecules and hydration layers [19]. However, a rigorous understanding of these effects and their direct contributions to enzymatic activity remains incomplete. Enzymes are proteins with catalytic functions and their highly regulated conformations are essential for their catalytic activity. To elucidate the effects of MW irradiation on enzymatic reactions, it is crucial to understand the interactions between MWs and proteins as well as how the resulting structural changes are linked to enzymatic function.

In this review, we summarize the fundamental effects of MW irradiation on proteins, with particular emphasis on how these effects relate to enzyme conformation and enzymatic reactions. In Section 2, we summarize studies examining the effects of MW irradiation on protein structure, including both experimental (wet lab) and computational (dry lab) approaches. Because few studies of this type have been conducted specifically on enzymes, research involving non-enzymatic proteins has also been included. In Section 3, we focus on enzymatic reactions in aqueous systems, which are considered suitable reaction environments for green chemistry, and summarize studies that have examined how MW irradiation influences enzymatic activity and reaction behavior. Because this field is still in its early stages of development, we present experimental directions for future research. The insights presented in this review are expected to contribute to a deeper understanding of MW-assisted enzymatic processes and the rational design of future sustainable reaction systems.

## 2. Effects of MWs on Protein Structure

The structures of proteins, including enzymes, have a hierarchical organization. The linear sequence of amino acids that make up the polypeptide chain forms the primary structure. Higher-order structures are formed through various interactions. Secondary structures result from local folding patterns primarily stabilized by hydrogen bonding along the polypeptide backbone, typically forming α-helices and β-sheets. These fundamental structural elements are arranged in a compact and functional conformation to form an overall three-dimensional tertiary structure. The structure at this level is stabilized by various interactions such as hydrophobic interactions, hydrogen bonds, ionic interactions, and disulfide bonds. The association of multiple folded polypeptide chains (subunits) produces complex quaternary structures that represent functional protein assemblies with properties distinct from those of individual monomers. In general, the biological functions of proteins, including enzymes, are expressed after achieving the correct three-dimensional conformation through proper folding.

Various spectroscopic and analytical techniques have been used to investigate the effects of MW irradiation on protein structures in aqueous solutions. Among them, circular dichroism (CD) spectroscopy is widely used to monitor changes in secondary structure content, such as α-helices and β-sheets. Infrared (IR) spectroscopy provides complementary information on the backbone conformation and hydrogen-bonding environments, particularly through amide I and II bands. Fluorescence spectroscopy is frequently used to probe tertiary structural changes by detecting alterations in the microenvironments of aromatic residues. To further characterize the higher-order structural changes and aggregation behavior, several complementary techniques were utilized. Ultraviolet–visible (UV–vis) spectroscopy can be used to detect changes in absorbance and turbidity associated with protein denaturation or aggregation. Dynamic light scattering (DLS) provides information on the hydrodynamic size and size distribution, enabling the assessment of MW-induced aggregation. Microscopic techniques such as atomic force microscopy (AFM) and transmission electron microscopy (TEM) offer direct visualization of protein morphology and aggregate structures at the nanoscale level. These techniques, often combined with thermal and kinetic analyses, enable the evaluation of MW-induced structural perturbations across different hierarchical levels of proteins.

The effects of MW on proteins in aqueous solutions are closely related to dielectric relaxation processes that occur over different frequency ranges (Table 1) [20]. These include α-dispersion, associated with counterion polarization and relaxation of electric double layer; β-dispersion, arising from rotational relaxation of protein molecules and interfacial polarization; δ-dispersion, attributed to the relaxation of hydration water surrounding the protein surfaces; and γ-dispersion, corresponding to the dipolar relaxation of bulk water. These relaxation processes are considered to influence protein behavior at different structural and dynamic levels under MW irradiation.

Here, we review studies examining the effects of MW irradiation on the hierarchical levels of the structures of proteins, including enzymes. Because few studies of this type have been conducted specifically on enzymes, research involving non-enzymatic proteins has also been included. We focused on experimental approaches using actual protein samples (Table 2) and computational simulations (Table 3).

### 2.1. Experimental Approaches Using Actual Proteins

#### 2.1.1. β-Lactoglobulin

β-Lactoglobulin, a globular protein composed of 162 amino acid residues, has been widely studied as a model system for protein folding and aggregation. In 2000, Bohr et al. [21] reported that MW irradiation promoted both refolding from cold-denatured states and denaturation of the native conformation of β-lactoglobulin, indicating that MWs can influence protein conformational equilibria. Subsequent studies examined MW-induced structural changes in this protein. In 2012, Hettiarachchi et al. [22] investigated MW-induced fibrillation of β-lactoglobulin. Under certain conditions, this protein can misfold and aggregate, forming amyloid fibrils that are characterized by a cross-β structure [23]. Heating a β-lactoglobulin solution (pH 2) at 80 °C for 2 h under MW irradiation led to pronounced self-assembly with a fibril yield comparable to that obtained after 16 h of conventional heating. Notably, prolonged MW irradiation beyond 2 h resulted in fibril degradation, suggesting that MW irradiation can both promote and disrupt fibril formation depending on the irradiation duration. Subsequently, in 2015, Lee et al. [24] demonstrated that varying the MW irradiation conditions, including irradiation time, intervals, and irradiation cycles, enabled control over fibril morphology, including helical pitch, diameter, and length. In a later study in 2018, Lee et al. [25] reported that the concentration of β-lactoglobulin influenced the formation and abundance of pre-fibrillar aggregates under MW irradiation. AFM analysis revealed that although the aggregate size remained relatively constant across concentrations of 0.1, 0.5, and 1 wt%, the number of aggregates increased in a concentration-dependent manner. These observations were consistent with the results obtained from Thioflavin T fluorescence assays, a widely used method for detecting amyloid fibrils.

#### 2.1.2. Bovine Serum Albumin

Bovine serum albumin (BSA) is a well-characterized globular protein widely used as a model system for investigating protein structure and stability. In 2003, Pomerai et al. [26] reported that 1 GHz MW irradiation promoted the formation of protein aggregates in aqueous BSA solutions, which was confirmed using DLS measurements showing increased scattering intensity in irradiated samples compared with non-irradiated controls; that is, MW irradiation promoted protein aggregation. In 2010, Calabrò et al. [27] investigated the effects of 900 MHz MW irradiation on the secondary structure of BSA using IR absorption spectroscopy and showed that the amide I band (approximately 1650 cm^−1^), which originates mainly from C=O stretching vibrations of peptide bonds, is highly sensitive to protein secondary structure. After 8 h of MW irradiation, an increase in the band intensity accompanied by a slight peak shift was observed, suggesting MW-induced structural changes. Fourier deconvolution analysis further revealed an increase in β-sheet content, indicating that the observed spectral shift was attributable to enhanced β-sheet formation.

#### 2.1.3. Bovine Insulin

Bovine insulin forms amyloid fibrils during its denaturation and aggregation [28]. In 2003, Pomerai et al. [26] used TEM to investigate the morphological effects of MW irradiation on bovine insulin. Treatment of an insulin solution (pH 2) with 1 GHz MW irradiation at 60 °C for 24 h resulted in pronounced amyloid fibril formation. In contrast, no fibrils were detected in samples subjected to the same thermal treatment in the absence of MW irradiation, indicating that MW irradiation played a critical role in promoting fibril formation under these conditions.

#### 2.1.4. Myoglobin

Myoglobin is a heme-containing protein predominantly found in the skeletal and cardiac muscles, where it functions as an intracellular oxygen reservoir. In 2004, Mancinelli et al. [29] investigated the effects of 1.95 GHz MW irradiation on myoglobin using UV–vis absorption spectroscopy to monitor heme-related spectral features. The spectral behavior of the MW-irradiated samples differed from that of the conventionally heated controls, suggesting that MW irradiation induced structural modifications in the vicinity of the heme group. Subsequently, in 2010, Calabrò et al. [27] examined changes in the secondary structure of myoglobin following MW irradiation at 900 MHz for 8 h using IR spectroscopy and showed that a shift of 2.5 cm^−1^ in the amide I band peak was observed after MW treatment, accompanied by an increase in band intensity. Fourier deconvolution analysis further revealed an increase in β-sheet content, indicating that MW irradiation induced alterations in the secondary structure of myoglobin.

#### 2.1.5. Globular Actin

Globular actin (G-actin) is a monomeric protein that polymerizes to form filamentous actin and plays a central role in cytoskeletal organization and cellular motility. In 2017, Lou et al. [30] investigated the effects of 2.45 GHz MW irradiation on the folding behavior of G-actin. The surface hydrophobicity and sulfhydryl group content measured in the samples afterMW irradiation reached a maximum at 300 W, indicating partial protein unfolding. Further increasing the MW power from 300 to 500 W led to a decrease in hydrophobicity and sulfhydryl content, suggesting the onset of a possible refolding transition.

#### 2.1.6. Acetylcholinesterase

Acetylcholinesterase is a key enzyme responsible for the rapid hydrolysis of the neurotransmitter acetylcholine at cholinergic synapses and neuromuscular junctions. In 2005, Vukova et al. [31] investigated the effects of 2.45 GHz MW irradiation for 30 min on acetylcholinesterase isolated from frog skeletal muscle. Enzyme activity assays revealed a decrease in enzymatic activity following MW irradiation, and IR spectroscopy showed an increase in β-sheet and random coil content; thus, MW irradiation is suggested to induce structural alterations that are associated with enzymatic function.

#### 2.1.7. Lysozyme

Lysozyme is a well-characterized antimicrobial enzyme that catalyzes the hydrolysis of β-(1,4)-glycosidic bonds in bacterial cell wall peptidoglycans. In 2010, Calabrò et al. [27] reported that MW irradiation of lysozyme for 8 h increased the intensity of the amide I band, suggesting alterations in its secondary structure. A decade later, in 2020, Yang et al. [32] evaluated the hydrolytic activity of MW-treated lysozyme and found that MW-irradiated samples exhibited higher enzymatic activity than those processed using conventional methods. In addition to enhanced activity, increased surface hydrophobicity and formation of lysozyme dimers were observed, indicating that MW irradiation induced conformational changes that may be associated with the observed enhancement of enzymatic function.

#### 2.1.8. Summary

Experimental studies investigating the effects of MW irradiation on various proteins have shown that MWs alter higher-order protein structures, often by inducing aggregation, fibrillation, or conformational changes. However, the detailed molecular mechanisms underlying MW-induced structural changes in proteins are not fully understood. Beyond the proteins discussed here, similar MW-induced effects have been reported in food-derived proteins, such as those from wheat and beef [33,34,35], highlighting the widespread interest in understanding MW–protein interactions.

**Table 2:** Experimental approaches using actual protein samples.

Protein	MW Frequency (GHz)	MW Irradiation Condition	Effect	Ref.
β-Lactoglobulin	2.45	800 W	Refolding from cold-denatured states	[21]
	2.45	800 W	Denaturation of native conformation	[21]
	2.45	4 W	Fibril formation under acidic conditions	[22]
	2.45	0.8–4 kJ (Input energy)	Fibril formation under acidic conditions	[24]
	2.45	1100 W	Aggregate formation under acidic conditions	[25]
BSA	1	0.5 W	Aggregate formation	[26]
	0.9	8–25 mA/m (Magnetic field strength)	Increase in β-sheet content	[27]
Bovine Insulin	1	0.5 W	Amyloid fibril formation under acidic conditions	[26]
Myoglobin	1.95	51 mW/g	Decrease in refolding rate under acidic conditions	[29]
	0.9	8–25 mA/m (Magnetic field strength)	Increase in β-sheet content	[27]
G-Actin	2.45	5.56–27.78 mW/cm^2^	Effect on folding	[30]
Acetylcholin esterase	2.45	10–20 mW/cm^2^	Increases in β-sheet and random coil content	[31]
Lysozyme	0.9	8–25 mA/m (Magnetic field strength)	Alterations in secondary structures	[27]
	2.45	270–630 W	Increased surface hydrophobicity and formation of lysozyme dimers	[32]

### 2.2. Simulation-Based Studies

Simulations of biomolecular behaviors, including those involving proteins, are typically computationally intensive and time-consuming. However, recent advances in computational techniques have significantly improved processing speed, enabling more detailed investigations of protein dynamics under various external stimuli. Importantly, simulation studies on MW effects differ substantially in their treatment of thermal contributions. In general, three modeling strategies can be identified: (i) isothermal simulations in which the temperature is maintained constant and MWs are represented as oscillating electric fields, thereby allowing the investigation of field-induced effects under controlled thermal conditions; (ii) non-equilibrium approaches that partially decouple or distinguish the response of different degrees of freedom to mimic MW-specific energy absorption prior to full energy redistribution; and (iii) thermally driven models in which MW irradiation is effectively interpreted as a heating source leading to temperature elevation. In this section, we review the representative studies within these frameworks (Table 3). Because few studies of this type have been conducted specifically on enzymes, research involving non-enzymatic proteins has also been included.

In 2020, Todorova et al. [36] investigated the effect of MW irradiation on apoC-II, a peptide with amyloidogenic potential, using all-atom non-equilibrium molecular dynamics (MD) simulations under isothermal conditions. In their model, the temperature was controlled using a thermostat, and the MW field was introduced as an external oscillating electric field, thereby isolating the field-induced effects. The conformational states of the peptides were found to depend on both the frequency and intensity of the applied electric field. At a high field strength of 7 × 10^8^ V/m, increasing the frequency from 1.0 to 5.0 GHz enhanced conformational diversity. At moderate field strength (3.85 × 10^7^ to 7 × 10^7^ V/m), the specific conformations were frequency-dependent: at 1.0 GHz, both fibril-promoting and fibril-inhibiting conformations coexisted, whereas at 2.5 GHz, fibril-promoting conformations predominated. At 5.0 GHz, the peptide exhibited increased molecular mobility with aromatic side chains oriented to support fibrillation and extended backbone structures. At low field strengths (7 × 10^5^ to 7 × 10^6^ V/m), hairpin-like conformations prone to amyloid formation were consistently observed across all frequencies.

In the same year, Gladovic et al. [37] used a non-equilibrium MD framework in which the MW-induced rotational excitation of water molecules was partially separated from the translational relaxation processes. This approach enables the investigation of MW-specific dynamic responses prior to complete energy redistribution. Their simulations revealed that MW irradiation weakened intermolecular hydrogen bonds between β-peptides and surrounding water molecules, resulting in significant conformational changes. Cluster analysis further indicated that MWs promoted the formation of hairpin structures, which are implicated in neurodegenerative diseases, such as Alzheimer’s, Parkinson’s, Huntington’s, and Creutzfeldt–Jakob diseases, as well as in cancer-related amyloidosis.

More recently, in 2024, Broz et al. [38] analyzed structural changes in 29 proteins under an MW field (2.51 × 10^3^ V/m) using isothermal MD simulations. The temperature was maintained constant, and MW effects were introduced via oscillating electric fields. These proteins tended to adopt more compact conformations under these conditions because of their enhanced intramolecular interactions, particularly the strengthened electrostatic interactions and hydrogen bonds.

In contrast, in 2025, Zhang et al. [39] combined spectroscopy and MD simulations to examine the thermal degradation of C-phycocyanin (CPC) under different heat-treatment conditions, including MW irradiation. Temperature effects were introduced via controlled heat-treatment protocols, and corresponding MD simulations were performed under elevated temperature conditions. Their results showed that MW fields at 10^8^ and 10^9^ V/m led to increased structural fluctuations and disrupted inter-residue interactions, which affected CPC conformation.

In 2018, Singh et al. [40] employed MD simulations to evaluate the effects of MW irradiation on hen egg-white lysozyme under isothermal conditions, modeling the MW field as an external oscillating electric field. Under continuous MW irradiation at a frequency of 10 GHz and an electric field strength of 8 × 10^2^ V/m, the displacement of 129 amino acid residues constituting the protein from their average positions was monitored. For irradiation times shorter than 15 min, negligible displacement was observed. However, discernible changes in the residue positions began to emerge at approximately 20 min, reached a maximum at 30 min, and then gradually decreased between 30 and 45 min. Complementary experiments on actual lysozyme samples, including CD and Raman spectroscopy, indicated no significant alterations in the secondary structure. These results suggest that, under the conditions studied, MW irradiation induces only minimal shifts in residue positions and has negligible effects on the secondary structure of the lysozyme.

In 2024, Tao et al. [41] used molecular docking and MD simulations to investigate the binding mechanism between transglutaminase (TGase) and its substrate under MW field conditions of 2.45 GHz. The MW field was modeled as an external oscillating electric field with varying intensities, and the temperature was maintained under isothermal conditions. Their results suggested that MW irradiation wasassociated with increased structural flexibility of TGase and partial conformational transitions from α-helices to turns or coils, which mightenhance the accessibility of substrate-binding sites and influence binding interactions.

In 2025, Ying et al. [42] investigated the impact of MW irradiation on fructan content in sourdough steamed cake and examined the mechanism of β-fructosidase FosE-substrate interactions using molecular docking and MD simulations. MW irradiation was effectively treated as a thermal condition, and corresponding simulations were performed to reflect temperature-dependent changes in enzyme–substrate interactions. Their results suggested that MW irradiation was associated with altered binding free energy and changes in Coulombic interaction patterns, which may influence the stability of the enzyme–substrate complex. Although structural changes in proteins and peptides have been reported at various electric field strengths based on MD simulations, in 2012, Damm et al. [43] conducted molecular mechanics simulations on trypsin and BSA and argued that conformational changes in proteins require electric field strengths of at least 1 × 10^7^ V/m.

In summary, simulation studies indicate that MW-induced effects on proteins and peptides are strongly dependent on key parameters such as electric field strength and frequency, leading to a range of reported structural responses, including conformational redistribution, compaction, and partial unfolding. A key limitation of current approaches is the inconsistent treatment of thermal effects. Many simulations model MW irradiation as an external electric field under isothermal conditions without explicitly accounting for the heat generation or thermoelectromagnetic coupling. Therefore, future work should employ multiphysics approaches that couple electromagnetic field propagation with heat transfer and MD to capture the interplay between field-induced polarization and local temperature increase [44,45]. Such frameworks are expected to provide a more realistic description of the MW–biomolecule interactions. It should also be noted that the electric field strengths employed in many simulation studies are significantly higher than those typically used in experimental MW systems. Although limited in number, simulation-based studies are expected to expand and contribute to a more comprehensive understanding of MW-induced structural changes in proteins.

**Table 3:** Computational approaches.

Protein/Peptide	MW Frequency (GHz)	MW Irradiation Condition	Effects of MW on Proteins	Ref.
apoC-II peptide	1.0, 2.5, 5.0	7 × 10^5^–7 × 10^8^ V/m	Increase in conformational diversity under high field strength Amyloid-prone hairpin-like conformations under low field strengths	[36]
β-peptide	– *	361–1730 W	Weakened intermolecular hydrogen bonds between the peptides and water	[37]
29 proteins	0.3–20	2.51 × 10^3^ V/m	Formation of more compact conformations	[38]
C-phycocyanin	2.45	10^5^, 10^8^–10^9^ V/m	Increased structural fluctuations and disrupted inter-residue interactions at 10^8^ and 10^9^ V/m	[39]
Lysozyme	10	800 V/m	Changes in residue positions	[40]
Transglutaminase	2.45	10^5^ V/m	Partial conformational transitions from α-helices to turns or coils	[41]
β-fructosidase FosE	2.45	10^4^–10^5^ V/m	Structural stability of the enzyme-substrate complex	[42]

* There is no description of the MW frequency.

## 3. Effects of MW Irradiations on Enzymatic Reactions

We reviewed both experimental and simulation-based studies demonstrating that MW irradiation can induce conformational changes in proteins. Because the protein structure is closely linked to its function, this section focuses on enzymes, which are proteins exhibiting catalytic activity. Although numerous studies on MW-assisted enzymatic reactions have been reported [46], here we specifically focus on enzymatic reactions in aqueous systems, which are regarded as suitable reaction media for green chemistry, and summarize the research examining how MW irradiation affects enzymatic activity and reaction behavior. To clarify the scope of this section, the included studies were limited to aqueous-phase enzymatic systems, in which MW effects can be directly compared with those under conventional heating under similar conditions. Studies without appropriate thermal controls or comparable experimental conditions were excluded to ensure consistency and interpretability.

### 3.1. Studies Focused on Enzyme Activity Derived from Product Yields

In 2008, Young et al. [47] investigated the effects of 2.45 GHz MW irradiation on the activity of β-glucosidase (Pfu CelB) from *Pyrococcus furiosus*, β-glucosidase (Pdu CelB) from *Prunus dulcis*, α-galactosidase (Tm GalA) from *Thermotoga maritima*, and carboxyesterase (SsoP1CE) from *Sulfolobus solfataricus* P1 (Figure S1). Enzyme activity was evaluated based on the product yield. MW irradiation increased Pfu CelB activity by approximately 10,000-fold, accompanied by a reduction in the optimal reaction temperature. Tm GalA and SsoP1CE showed approximately 10-fold increase in activity, whereas Pdu CelB showed no enhancement, indicating that MW-induced activation is enzyme-specific.

In 2014 and 2023, Nagashima et al. [48,49] investigated the hydrolysis of 4-methoxyphenyl β-D-glucopyranoside catalyzed by a thermostable β-glucosidase HT1 (optimal temperature: 60 °C). They observed that MW irradiation at 0.4 and 2.45 GHz enhanced the reaction compared with that under conventional heating at the same temperature, whereas no enhancement was noted at 5.8 GHz. Furthermore, under 0.4 and 2.45 GHz irradiation, the optimal temperature for the enzyme decreased to 50 °C, representing a 10 °C reduction relative to that under conventional heating. Dielectric measurements suggested that at 0.4 GHz, ionic conduction loss occurred; at 2.45 GHz, both ionic conduction loss and water dielectric loss occurred; whereas at 5.8 GHz, only water dielectric loss was observed. These results imply that MWs at 0.4 and 2.45 GHz interact with ions, potentially affecting enzyme–substrate affinity during complex formation. However, the study did not include a detailed analysis using the Michaelis–Menten equation.

In 2015, Mazinani et al. [50] investigated the trypsin-catalyzed hydrolysis of casein under 2.45 GHz MW irradiation (Figure S2). Enhanced enzymatic activity was observed at high substrate concentrations. CD spectroscopy confirmed MW-induced alterations in the secondary structure of trypsin, suggesting that these structural changes may have induced its activity. In contrast, in 2016, Mazinani et al. [51] reported no significant effect of 2.45 GHz MW irradiation on the activity of α-amylase (starch hydrolysis) or alkaline phosphatase (p-nitrophenyl phosphate hydrolysis) compared with that under conventional heating.

These studies primarily evaluated enzyme activity based on the product yield, offering limited insights into the mechanistic basis of MW-induced enhancement. In 2018, Cao et al. [52] focused on the structural changes in TGase and examined the effects of MWs on its activity. MW irradiation enhanced TGase activity, and UV–vis and fluorescence spectroscopy revealedconformational changes. CD spectroscopy revealed decreased α-helix content and increased β-sheet and β-turn structures, which are changes that were not observed with conventional water bath heating. Although MW-induced structural modifications have been confirmed, the precise mechanism by which they influence enzymatic activity remains unclear.

The studies in this section focused on assessing enzyme activity primarily based on product yield. To gain a deeper understanding of the MW effects, such yield-based evaluations should be complemented by structural analyses and kinetic studies using the Michaelis–Menten equation.

### 3.2. Detailed Studies Utilizing the Michaelis–Menten Equation

In 2009, Pavelkic et al. [53] investigated the effects of 2.45 GHz MW irradiation on porcine pepsin. Kinetic analysis using the Michaelis–Menten equation revealed that compared with those under conventional heating, MW irradiation decreased V_max_ and increased K_m_. As K_m_ reflects the affinity of the enzyme–substrate complex, these results suggest that MW irradiation reduces substrate binding. In 2018, Rokhati et al. [54] studied the effects of MW irradiation on the hydrolysis of chitosan by cellulase. Michaelis–Menten analysis revealed that V_max_ under MW irradiation was approximately 20 times higher than that under conventional heating, whereas K_m_ decreased, indicating that MW irradiation increased the affinity between the enzyme and its substrate. Because K_m_ reflects the affinity of the enzyme–substrate complex, this result impliedthat MW irradiation enhanced substrate binding.

Incorporating Michaelis–Menten kinetics allows for a more mechanistic evaluation of the MW effects. Although MW-induced changes in enzymatic activity have been reported in numerous studies, few studies have included detailed kinetic analyses. Future studies should integrate the kinetic parameters with structural analyses (discussed in Section 2) to clarify how MW irradiation affects enzymatic reactions at the molecular level.

Although the molecular-level mechanisms underlying the effects of MW irradiation remain largely unclear, the application of MW irradiation to enzymatic processes is widely used in the food industry [18,55,56,57,58], and MW-assisted techniques have been utilized to control enzyme activity, accelerate enzymatic hydrolysis, and enhance extraction processes involving enzyme-mediated reactions. Future research in this field is expected to provide further mechanistic insights and practical benefits.

## 4. Conclusions

As illustrated in this review, MW irradiation can influence higher-order structures of proteins and induce phenomena such as fibrillation and aggregate formation. MD simulations have demonstrated that MW irradiation causes model peptides and proteins to adopt conformations prone to aggregation. Although structural changes in proteins under MW irradiation are becoming increasingly evident, the detailed mechanisms by which MWs affect enzymatic activity are still under investigation. To elucidate these mechanisms, it is essential to go beyond assessing changes in reaction yields and integratekinetic analyses based on the Michaelis–Menten equation with structural studies, including nuclear magnetic resonance spectroscopy and X-ray crystallography. Furthermore, a more systematic consideration of the dielectric properties and frequency-dependent responses of each component involved in the enzymatic reactions, together with simulation techniques, will be increasingly important for evaluating the effects of MW irradiation. In particular, linking dielectric and ionic conduction losses to molecular relaxation processes is important for a more physically grounded interpretation of frequency-dependent MW effects. Multiphysics modeling that couples electromagnetic field distributions with heat transfer can also be incorporated to account for the nonuniform heating effects. In addition, standardized and comparable experimental parameters, including irradiation conditions and dosimetric parameters, such as electric field strength and specific absorption rate, are crucial for improving cross-study comparability. However, detailed information on the MW irradiation equipment and operating conditions is often insufficiently reported, which limits rigorous comparisons across studies. We also note concerns regarding the robustness of the kinetic data, including the limited reporting of replicates, isothermal control, diffusion effects, and uncertainties. Future MW-based Michaelis–Menten analyses should, therefore, follow best practices, including rigorous temperature control, adequate replication, and transparent reporting of experimental uncertainties. Therefore, future studies should adopt an integrated approach that combines appropriate control experiments, such as conventional or alternative heating methods, standardized experimental parameters, and multiphysics modeling techniques. Such an approach will enable a more reliable and comprehensive evaluation of MW-specific effects, thereby improving the mechanistic understanding and supporting the rational design of MW-assisted enzymatic processes.

## Figures and Tables

**Table 1:** Frequency-dependent dielectric dispersion of aqueous protein solutions.

Frequency	Dispersion	Main Contributing Species	Physical Origin
Hundreds of Hz–tens of kHz	α-dispersion	Ion	Counterion polarization and relaxation of electric double layer
Hundreds of kHz–hundreds of MHz	β-dispersion	Protein	Rotational relaxation of protein molecules and interfacial polarization
Hundreds of MHz –several GHz	δ-dispersion	Hydration water	Relaxation of hydration water at protein surfaces
Several GHz–hundreds of GHz	γ-dispersion	Bulk water	Rotational relaxation of free water molecules

## Data Availability

No new data were created or analyzed in this study. Data sharing is not applicable to this article.

## Supplementary Materials

The following supporting information can be downloaded at: https://www.mdpi.com/article/10.3390/molecules31111843/s1, Figure S1: Hydrolysis of 4-nitrophenyl β-D-glucopyranoside catalyzed by a β-glucosidase CelB under microwave irradiation; Figure S2: Hydrolysis of Nα(±)-benzoyl-D/L-arginine 4-nitroanilide hydrochloride by trypsin under microwave irradiation.
