# Supplementary material for: Effect of Microwave on Protein Conformations and Enzymatic Reactions
Source: Molecules. 2026 May 27;31(11):1843. doi: 10.3390/molecules31111843 (PMC13257693; doi:10.3390/molecules31111843)
Supplement: Supplementary file 1 [file molecules-31-01843-s001.zip › molecules-4182900-supplementary.pdf]

Supplementary Materials

Effect of Microwave on Protein Conformations and Enzymatic Reactions

Fumihiro Kayamori [1,2,*] and Kenji Usui [1,2,3]

[1] Faculty of Frontiers of Innovative Research in Science and Technology (FIRST), Konan University, 7-1-20 Minatojima-minamimachi, Chuo-ku, Kobe 650-0047, Hyogo, Japan

[2] Research Institute for Nanobio-Environment and Non-Ionizing Radiation (RINNIR) Konan University, 7-1-20 Minatojima-minamimachi, Chuo-ku, Kobe 650-0047, Hyogo, Japan

[3] Beyond5G, Donated Lectures, Konan University, 8-9-1 Okamoto, Higashinada-ku, Kobe 658-8501, Hyogo, Japan

Contents

Figure S1. Hydrolysis of 4-nitrophenyl β-D-glucopyranoside catalyzed by a β-glucosidase CelB under microwave irradiation.

Figure S2. Hydrolysis of Nα(±)-benzoyl-D/L-arginine 4-nitroanilide hydrochloride by trypsin under microwave irradiation.

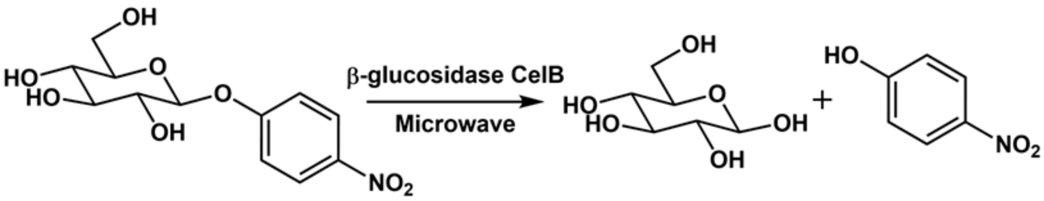

Figure S1. Hydrolysis of 4-nitrophenyl β-D-glucopyranoside catalyzed by a β-glucosidase CelB under microwave irradiation.

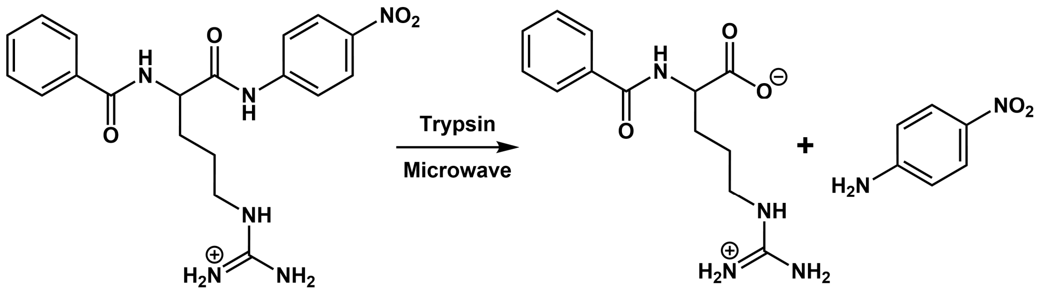

Figure S2. Hydrolysis of Nα(±)-benzoyl-D/L-arginine 4-nitroanilide hydrochloride by trypsin under microwave irradiation.
